# Genome-Wide Screen of DNA Methylation Changes Induced by Low Dose X-Ray Radiation in Mice

**DOI:** 10.1371/journal.pone.0090804

**Published:** 2014-03-10

**Authors:** Jingzi Wang, Youwei Zhang, Kai Xu, Xiaobei Mao, Lijun Xue, Xiaobei Liu, Hongjun Yu, Longbang Chen, Xiaoyuan Chu

**Affiliations:** 1 Department of Medical Oncology, Jinling Hospital, Nanjing Clinical School of Southern Medical University, Nanjing, P.R. China; 2 Department of Medical Oncology, Yangzhou No. 1 People's Hospital, The Second Clinical School of Yangzhou University, Yangzhou, P.R. China; University of Navarra, Spain

## Abstract

Epigenetic mechanisms play a key role in non-targeted effects of radiation. The purpose of this study was to investigate global hypomethylation and promoter hypermethylation of particular genes induced by low dose radiation (LDR). Thirty male BALB/c mice were divided into 3 groups: control, acutely exposed (0.5Gy X-rays), and chronic exposure for 10 days (0.05Gy/d×10d). High-performance liquid chromatography (HPLC) and MeDIP-quantitative polymerase chain reaction (qPCR) were used to study methylation profiles. DNMT1 and MBD2 expression was determined by qPCR and western blot assays. Methylation and expression of Rad23b and Ddit3 were determined by bisulfate sequencing primers (BSP) and qPCR, respectively. The results show that LDR induced genomic hypomethylation in blood 2 h postirraditaion, but was not retained at 1-month. DNMT1 and MBD2 were downregulated in a tissue-specific manner but did not persist. Specific hypermethylation was observed for 811 regions in the group receiving chronic exposure, which covered almost all key biological processes as indicated by GO and KEGG pathway analysis. Eight hypermethylated genes (Rad23b, Tdg, Ccnd1, Ddit3, Llgl1, Rasl11a, Tbx2, Scl6a15) were verified by MeDIP-qPCR. Among them, Rad23b and Ddit3 gene displayed tissue-specific methylation and downregulation, which persisted for 1-month postirradiation. Thus, LDR induced global hypomethylation and tissue-specific promoter hypermethylation of particular genes. Promoter hypermethylation, rather than global hypomethylation, was relatively stable. Dysregulation of methylation might be correlated with down-regulation of DNMT1 and MBD2, but much better understanding the molecular mechanisms involved in this process will require further study.

## Introduction

Low dose radiation (LDR) sources are nearly ubiquitous in our environments owing to nuclear tests, radioactive waste, radiation accidents, and diagnostic, therapeutic and occupational exposures. It is widely believed that continuous exposure to LDR is a major cause of chronic human diseases, including cancer [1]. Non-targeted effects of radiation contributed to mutagenesis and carcinogenesis are often grouped as bystander effects and radiation-induced genomic instability (RIGI) [2]. RIGI is observed in cells at delayed times after exposure and sometimes manifests in their progeny over many generations [3]. RIGI mechanisms have been studied extensively in clones displaying genomic instability developed in the Morgan laboratory. Alterations that involve DNA sequence, such as radiation-induced mutations or double-strand breaks alone, or changes in messenger RNA (mRNA) levels, do not account for the initiation or perpetuation of RIGI in these clones, [4], [5]. Therefore, inherited epigenetic changes that control cellular phenotype without directly altering DNA sequence may be involved.

Epigenetics can play an important role in many cellular processes from gene expression to cellular proliferation and an aberrant epigenetic state of a cell can lead to carcinogenesis [6]. The most extensively studied epigenetic mechanism is DNA methylation. It has been suggested that changes in genomic DNA methylation post-irradiation may be correlated with initiation of genomic instability [7], [8]. These data provide a link among radiation exposure, DNA methylation and carcinogenesis. However, the influence of radiation exposure on DNA methylation, especially local hypermethylation at gene promoters, is still poorly understood. Thus, in this study, we investigated global DNA hypomethylation and promoter hypermethylation of particular genes in BALB/c mice after LDR (0.5Gy) treatment.

## Methods

### Irradiation of animals

Thirty males BALB/c mice (5 weeks old) were purchased from the Academy of Military Medical Science(Beijing, China), kept in the Comparative Medicine Department of Jinling Hospital (Nanjing, China). Mice were bred under controlled temperature and humidity, with a 12-hour light/dark cycle and sterile food and water ad libitum. Animal studies were carried out in strict accordance with guidelines for the Care and Use of Laboratory Animals of the Jinling Hospital. The protocol was approved by the Animal Ethics Committee of Jinling Hospital (No. 2010062412). All efforts were made to minimize suffering. We established three animal models in the present study: control, acutely exposed, and chronic (fractional) exposure to X-rays. Mice from the ‘fractional/chronic’ group were exposed to whole body irradiation of 0.5Gy applied as 5cGy of X-rays per day for 10 days, to mimic chronic repetitive exposure [9]. The ‘acute’ group received 0.5Gy of X-rays in single dose on the 10 th day of treatment of the ‘chronic’ group. Control mice were sham treated. To determine the early effect of irradiation, 15 animals were divided into 3 groups and sacrificed 2 hours after the last irradiation. The other 15 animals were sacrificed 1 month later (delayed effect). Following sacrifice, blood, kidney, liver, spleen, brain and lungs were collected. All tissues were divided into two equal parts. One part was placed into the Trizol (Invitrogen, Life Technologies, Foster City, CA, USA) solution and the second part frozen and used for DNA extraction.

### High-performance liquid chromatography (HPLC)

Global DNA methylation was detected by HPLC [10]. Firstly, we used the One-Step DNA Hydrolysis Kit (Epigentek, Farmingdale, NY, USA) to rapidly hydrolyze DNA to deoxynucleosides in a single incubation. Products were created using an Agilent Series 1200 liquid chromatograph equipment and a XB-C18 (150×4.6 mm, 5 µm, Welch Materials, Shanghai, China) column. Standard curve was drawed, linear equations of dC and 5mdC were established according to the peak area - mass concentrations. Methylation levels were calculated according to the formula: 5mdC% = 5mdc/(dC+5mdC)×100%.

### Quantitative polymerase chain reaction (qPCR)

Total RNA was isolated using TRIzol reagent (Invitrogen). Reverse transcription (RT) was performed using 2 µg of total RNA with a first strand cDNA kit (Takara, Shiga, Japan), according to the manufacturer's instructions. PCR amplification was performed for 20 s at 95°C, followed by 40 cycles at 95°C for 5 s, and annealing/extension at 60°C for 30 s in an ABI 7300 Thermocycler (Applied Biosystems, Foster City, CA, USA), using the SYBR Premix Ex Taq kit (Takara). The specific primer sequences for each gene are listed in **Table S1**. Data analysis was done using the 2^−ΔΔCT^ method for relative quantification. All samples were normalized to GAPDH.

### Western blotting

Cell protein lysates were separated in 10% SDS-PAGE, and electroblotted on to PVDF membranes. After blocking with buffer containing 5% low fat milk and 0.1% TBST, membranes were incubated with DNMT1, MBD2, or actin antibody (goat polyclonal IgG; Santa Cruz Biotechnology, Dallas, TX, USA) and secondary antibody (donkey anti-goat IgG; Santa Cruz). Finally, results were photographed with ECL substrate.

### MeDIP-on-chip and and Analysis

The genomic DNA extracted from whole blood samples was examined using MeDIP-on-chip (Nimblegen, Madison, WI, USA). Experimental details are listed in **Methods S1**.

### Real-time PCR on MeDIP-enriched DNA

We carried out real-time PCR with 20 ng immunoprecipitated methylated DNA and one-fifth of input DNA to verify MeDIP-chip results [11]. The ABI PRISM7900 System (Applied Biosystems) and 2×PCR master mix (Superarray, Houston, TX, USA) were used to perform real-time PCR. Primers used are listed in **Table S1**. All reactions were done in duplicate, and standard curves were calculated on serial 10-fold dilutions (from 1 to 1×10^−6^) of input genomic DNA. Signal ratios of immunoprecipitated DNA *vs* input DNA were calculated as a measure for representing the relative enrichment of methylation in a particular sample.

### Bisulfite sequencing (BSP)

After genomic DNA extraction and spectrophotometric quantization, 1 µg of genomic DNA was bisulfite-treated with EZ-DNA methylation Gold Kit (Zymo Research, Irvine, CA, USA). Bisulfite treated DNA was amplified by PCR with BSP primers, using sequences designed by Methyl Primer Express Software v1.0 and listed in **Table S1.** PCR products were cloned into the pUC57 vector (Genscript, Nanjing, China), and five clones selected and sequenced from each sample.

### Statistical analysis

The SPSS 13.0 software systerm (SPSS, Chicago, IL) was used for statistical analysis. Data are expressed as the mean±standard error. The differences between groups were analyzed using a Student t test when only 2 groups or 1-way analysis of variance (ANOVA) when more than 2 groups were compared. Differences were considered statistically significant at *P*<0.05.

## Results

### Effect of LDR-induced Global methylation changes in blood

**Figure 1:**
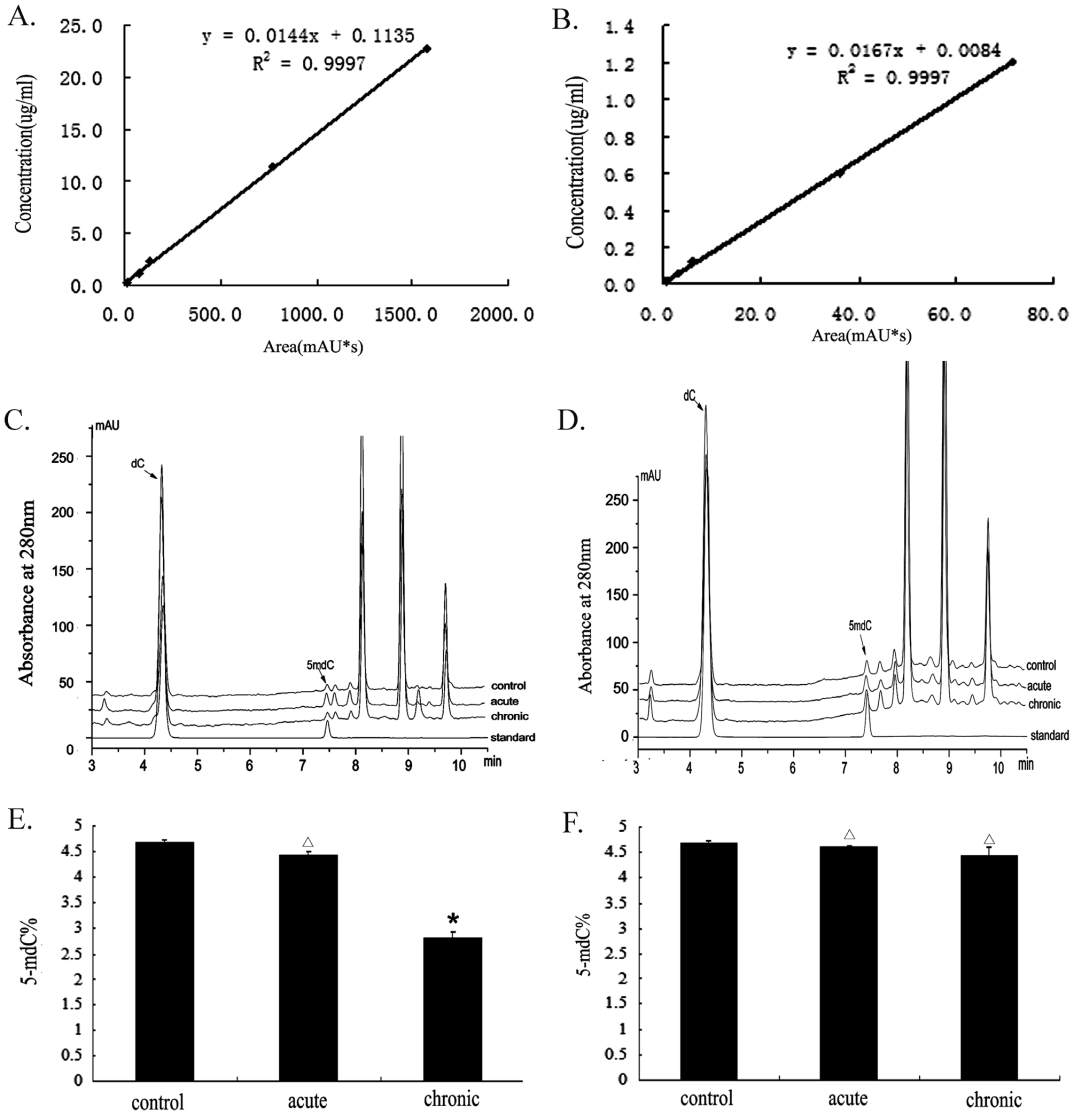
Effects of low dose X-rays on global methylation levels in mouse blood. Whole blood was sampled 2(dC and 5-mdC) detected by HPLC. Standard curve of dC(A) and 5mdC (B). DNA hydrolyzate compared with standard by liquid chromatography, early effects (C) and delay effects (D). Global methylation represented by 5-mdC(%), early effects (E) and delay effects (F). *P<0.05 versus control. ^△^ *P*>0.05 versus control.

Contents of dC and 5mdC in DNA hydrolyzate were calculated according to the standard curve. Chronic LDR exposure resulted in significant loss of DNA methylation compaired to control and acute exposure (*P*<0.05), 5-mC content in control, acute and chronic exposure was 4.67±0.17%, 4.43±0.13% and 2.82±0.31%, respectively. Changes previously observed at 2 h upon chronic exposure, faded completely after one month (Figure 1).

### Role of DNMT1 and MBD2 in radiation-induced alterations on DNA methylation patterns

**Figure 2:**
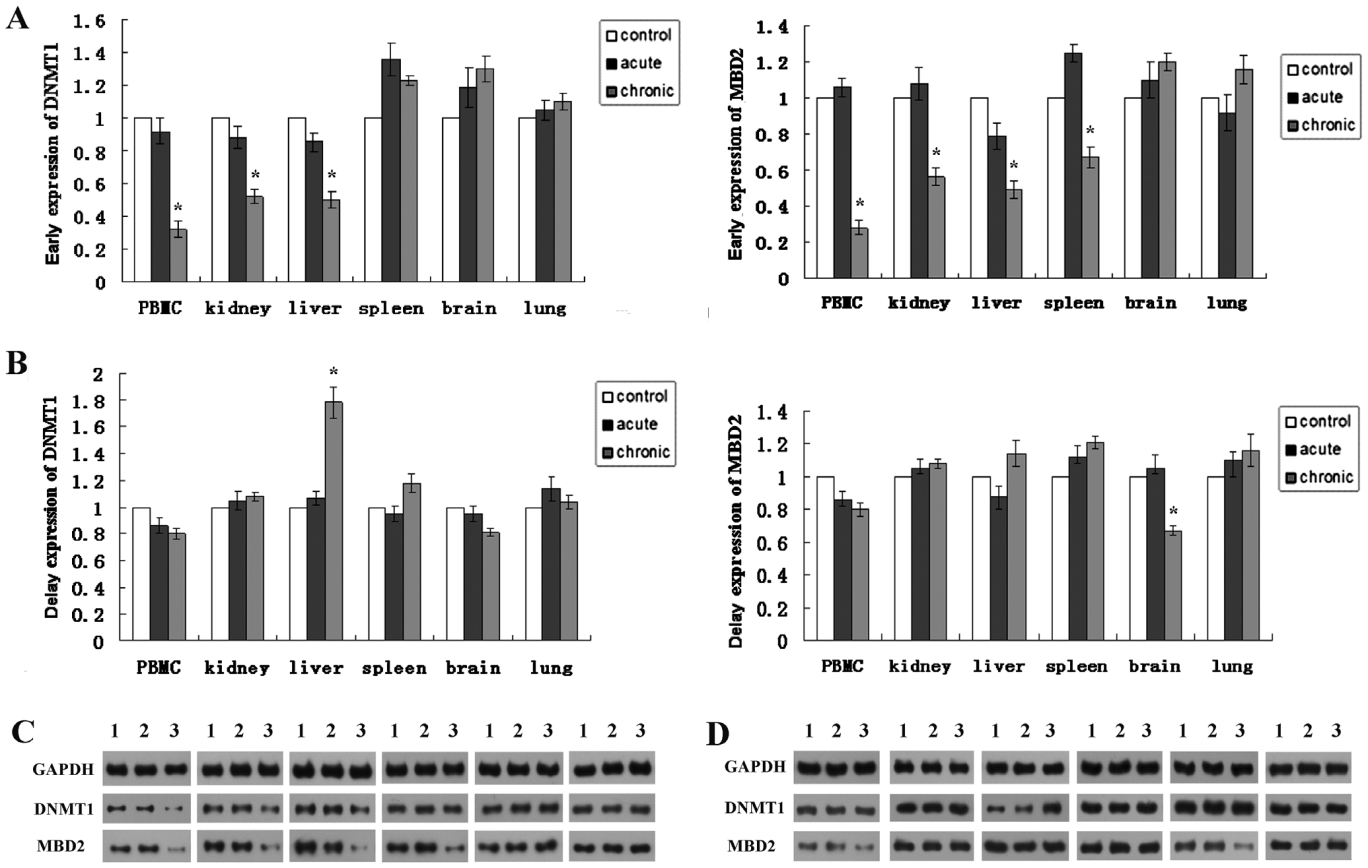
DNMT1 and MBD2 expression determined by realtime-PCR (A,B) and Western blot (C,D). Data are representative of at least 3 independent experiments. Early effects mean 2(A,C); Delay effects mean 1 month postirradiation (B,D). PBMC, peripheral blood mononuclear cell. 1, control; 2, acute exposed to 0.5 Gy; 3, chronic fractionated exposure. ^*^ *P*<0.05 versus control.

We evaluated the effects of acute and chronic LDR on the expression of DNMT1 and MBD2 in various tissues. Both of DNMT1 mRNA and protein expression were significantly downregulated in chronically exposed peripheral blood mononuclear cells (PBMC), kidney and liver tissues (*P*<0.05, **Figure 2A,B**). Similarly, a pronounced decrease of the expression of MBD2 could be found in PBMC, kidney, liver and spleen (*P*<0.05, **Figure 2A,B**). However, no changes were observed in the acutely exposed group. Delay effect changes in gene expression were not observed in groups receiving acute or chronic exposure. The exception was DNMT1 upregulation in chronically exposed liver, and downregulation of MBD2 in chronically exposed brain (*P*<0.05, **Figure 2** **C,D**).

### GO enrichment and KEGG pathway analysis of differential genome-wide promoter methylation regions in chronically irradiated mice

**Figure 3:**
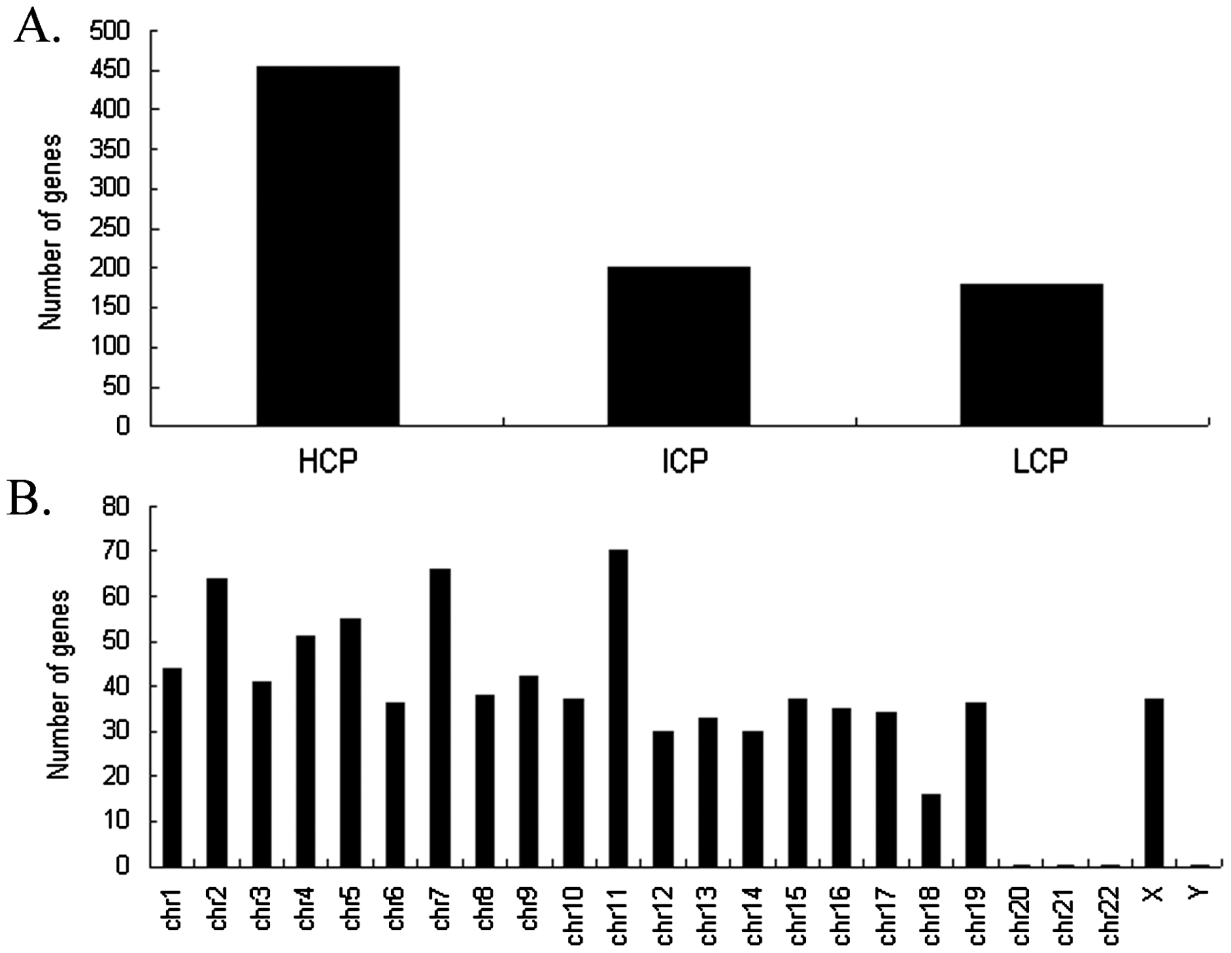
Distribution of 811 hypermethylated genes that showed hypermethylatin in chronic exposed group, but no methylation in control and acute group. (A) The promoters are subdivided into three classes based on CpG ratio, GC content and length of CpG-rich region: high (HCP), low (LCP), and intermediate (ICP). (B) Chromosome location.

**Figure 4:**
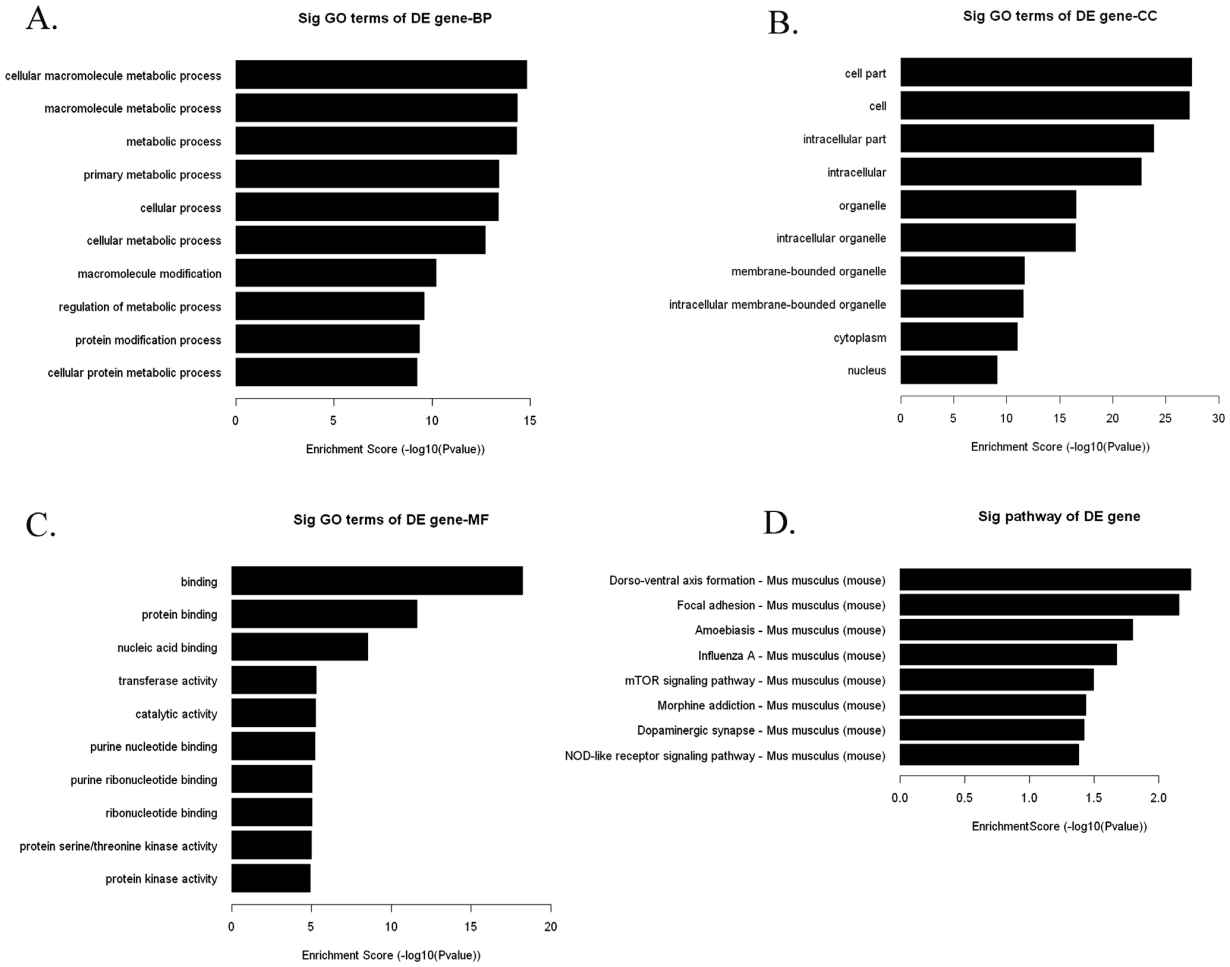
Functional and pathway analysis of the 811 genes with hypermethylated promoter identified by MeDIP-chip. Gene ontology (GO) analysis by three domains: Biological Process (A), Cellular Component (B) and Molecular Function (C). (D) KEGG Pathway analysis.

Genomic DNA extracted from whole blood samples from all three groups were evaluated for methylation using MeDIP-on-chip. We filtered the enrichment from the control and acute exposure group separately, and focused on signals enriched in chronic group. Therefore, 811 regions were selected because they exhibited hypermethylatin in chronic exposed group and unmethylation in control and acute group. Chromosomal locations of the 811 hypermethylated regions and their classification based on CpG content (LCP  =  low CpG content, ICP  =  intermediate CpG content, and HCP  =  CpG rich) are presented in **Figure 3**. In order to identify functions associated with these differently methylated CpGs and genes, we performed gene ontology (GO) analyses by three domains: Biological Process, which included metabolic process, cellular process, protein modification process, regulation of gene expression, developmental process, cellular component organization or biogenesis, etc. (**Table S2**); Cellular Component, which were mostly cell part and organelle (**Table S3**); Molecular Function, which were mainly related to protein binding, nucleic acid binding, transferase activity, catalytic activity, etc. (**Table S4**). KEGG Pathway analysis contained Dorso-ventral axis formation, Focal adhesion, mTOR signaling pathway, NOD-like receptor signaling pathway, etc. (**Figure 4**).

### Validation of the methylation status in the gene promoter by MeDIP-qPCR

In the MeDIP-chip array, the promoters are subdivided into three classes based on CpG ratio, GC content and length of CpG-rich region: (HCP, LCP, and ICP). Only ICPs and HCPs demonstrated association with methylation, whereas genes under the control of LCPs were expressed independently of methylation status [12]. We then used proximal methylation level, promoter CpG content (HCP or ICP), peak score and peak M value to select methylation marker. Eight methylated genes confirmed in chronic exposure samples were selected for further analysis (**Table S5**). MeDIP-qPCR was performed to evaluate their methylatoin status. All of 8 candidate genes (Rad23b, Tdg, Ccnd1, Ddit3, Llgl1, Rasl11a, Tbx2, Scl6a15) were confirmed to be hypermethylated in chronic exposure samples (**Table 1**). Thus, the accuracy of methylation chip in our study was credible.

**Table 1:** Real-time PCR on MeDIP-enriched DNA^a^.

Group	Rad23b	Tdg	Ccnd1	Ddit3	Llgl1	Rasl11a	Tbx2	Slc6a15
control	0.032	NA	0.069	0.049	NA	NA	NA	4.95E-04
acute	0.040	NA	0.098	0.250	NA	NA	NA	8.07E-04
chronic	1.220*	0.988*	2.987*	1.395*	1.117*	3.381*	0.507*	1.018*

a The data were the mean ratios of the signals in the immunoprecipitated DNA *vs* input DNA.

* *P*<0.05 versus control and acute exposure group.

NA, no amplification.

### Methylation and expression of candidate genes in irradiated tissues

**Figure 5:**
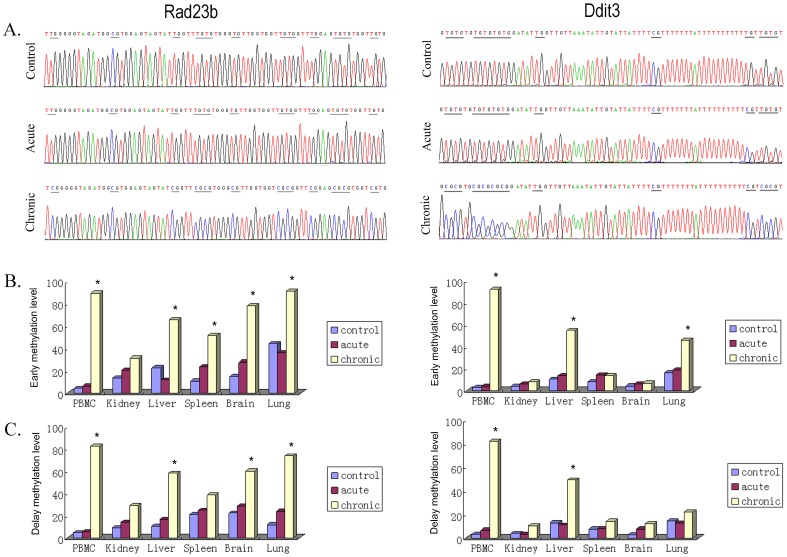
Rad23b and Ddit3 methylation determined by BSP. Bisulfite treated DNA was amplified by PCR with BSP primers. PCR products were cloned into the pUC57 vector, and five clones selected and sequenced from each sample. Methylation level was defined as the ratio of methylated CpG sites in all clones. (A) Typical sequencing results; (B) early effects, tissues were collected 2 h postirradiation; (C) delay effects, 1 month postirradiation. ^*^ *P*<0.05 versus control.

To test whether the impact of radiation on promoter methylation was consistent with the results of the peripheral blood, Rad23b and DNA-damage-inducible transcript 3 (Ddit3) genes were further evaluated for methylation status in various tissues using BSP. Relative to controls and the acute exposure group, both Rad23b and Ddit3 showed hypermethylation in PBMC following chronic exposure, which was consistent with MeDIP-qPCR results. Also, Rad23b was hypermthylated in liver, spleen, brain and lung, Ddit3 was hypermthylated in liver and lung in the chronic exposure group, for which samples were collected 2 h after the last irradiation (early effects). Furthermore, except for Rad23b methylation in spleen and Ddit3 methylation in lung, these changes persisted for 1 month postirradiation (delay effects), including in blood (**Figure 5**) (**Figure S1**).

**Figure 6:**
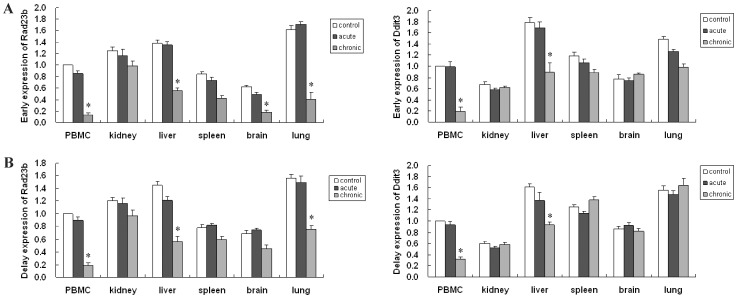
Rad23b and Ddit3 expression determined by realtime-PCR. Data analysis was done using the 2^-ΔΔCT^ method for relative quantification. The expression data was normalized to blood sample in control group. (A) early effects; (B) delay effects. ^*^ *P*<0.05 versus control.

Next, qPCR was used to detect Rad23b and Ddit3 mRNA expression in irradiated tissues. In general, Rad23b or Ddit3 expresison was downregulated in specific tissue which was hypermethylated, whenever the early or delay time postirradiation (**Figure 6**). Thus, changed DNA methylation indeed changes the expression of these genes.

## Discussion

Exposure to ionizing radiation may result in the manifestation of a variety of deleterious genetic as well as epigenetic effects. Methylation is one of the most important epigenetic mechanisms implicated in human carcinogenesis and in the maintenance of the genomic instability [13]. Attempts to detect LDR-induced methylation have been hampered by the lack of consistent results from methylation analyses. Early work demonstrated that four different cell lines exposed to 0.5–10 Gy of ^60^ Co γ rays exhibited hypomethylation 1–3 days postirradiation [14]. Another group later evaluated one of these same cell lines using 10 Gy of X rays and observed no change in DNA methylation over the same 3-day postirradiation time [15]. More recently, predominantly hypermethylation and specific hypomethylation were observed 20 population doublings after irradiation of cultured human keratinocytes using 0.1–1 Gy of ^60^ Co γ rays [16]. The variability in these findings might be explained by the use of different radiation doses or dose rates, cell lines or postirradiation times.

The effects of radiation on DNA methylation have also been evaluated *in vivo*. A significant decrease in global methylation as well as the accumulation of DNA damage was observed in the thymus of mice exposed to 0.5 Gy X-ray fractionated irradiation. Altered DNA methylation was associated with reduced expression of maintenance (DNMT1) and *de novo* DNA methyltransferase DNMT3a/3b. Irradiation also resulted in reduction of methyl-binding proteins MeCP2 and MBD2 [17]. Another study found muscle hypomethylation after chronic X-ray exposure, but no effect on liver or muscle global methylation 2 h after acute X-ray exposure [18]. On the basis of these studies, our study confirmed the finding that chronic LDR lead to a significant loss of DNA methylation in whole blood from irradiated mice, along with decreased DNMT1 and MBD2 expression in PBMC, which might account for genomic hypomethylation in blood [19]. Further, DNMT1 and MBD2 expression showed a tissue-specific pattern in different tissues from irradiated mice, which might be related to previous results that chronic LDR induced tissue-specific hypomethylation changes [9].

Genome-wide DNA hypomethylation is often viewed as a sign of genome destabilization, associated with chromosomal and genomic instability, and increased mutation rates [20]. This phenomenon is often observed in various cancers and at pre-cancerous stages. In this study, we also evaluated the persistence of the DNA hypomethylation following irradiation exposure. However, hypomethylation changes of blood in chronic LDR group had been repaired after 1 month. Moreover, expect for MBD2 downregulation in PBMC, decreased DNMT1 and MBD2 expression had been restored. In addition, DNMT1 was upregulated in liver and MBD2 downregulated in brain, which was not apparent early on. This may be due to differential radiosensitivity between tissues, and different roles that methylation-associated proteins play in different tissues. It may be that the body had adaptive capacity to repair DNA damage in the intermittent period of radiation, thus restoring genomic hypomethylation [21]. Once damage repair fails to complete in some radiosensitive tissues, RIGI may be maintained, the potential for carcinogenesis increased [22].

Local promoter methylation is an important feature of cancer, and one of the main effects caused by radiation. For example, LDR significantly increased DNA methylation at the yellow agouti (A^vy^) locus in the A^vy^ mouse model [23]. Aberrant promoter hypermethylation of p16 and RASSF1A has also been observed in ultraviolet (UV)-exposed skin and UV-induced skin tumors of mice [24]. However, promoter methylation induced by LDR has not been systematically studied. We ultilized MeDIP-on-chip to profile the promoter methylation of whole blood, and selected 811 regions with hypermethylation in chronic exposure group. These regions are normally distributed across almost all chromosomes, except for chromosomes 20, 21, 22, and Y. GO and KEGG pathway analysis revealed that they covered almost all key biological processes. Therefore, chronic LDR has an extensive and profound impact on gene promoter hypermethylation.

Eight methylated genes in chronic exposure samples were selected for further analysis. These genes were involved in DNA repair, cell cycle, apoptosis, hippo signaling pathway, GTP catabolic process, heart development and intracellular transport. MeDIP-qPCR results confirmed the accuracy of our methylation chip data, and suggested that 8 genes might exert crucial roles in radiation biology. Among them, Rad23b, also known as hHR23B or P58, a nucleotide excision repair gene, plays a key role in DNA damage recognition and repair [25]. Ddit3 is expressed ubiquitously and is induced by a wide variety of treatments such as DNA lesion, hypoglycaemia, radiation and cellular stress. Previous studies have confirmed the role of Ddit3 in regulating of cellular growth, differentiation and apoptosis, and inhibiting the G1 to S phase transition [26], [27]. These two genes, which are involved in typical biological processes of DNA damage, have been reported to be regulated by DNA methylation. They also displayed tissue-specific hypermthylation and downregulation following chronic exposure. It is noteworthy that most of these changes were sustained after 1 month. We hypothesize that promoter hypermethylation is relatively stable conformation, which indeed changes the expression of key genes and influence LDR-induce pathogenesis to a greater extent.

Apart from tumor suppressor genes, some oncogenes, such as Cyclin D1 (Ccnd1), also displayed hypermethylation in blood exposed to LDR, wherein its epigenetic silencing might provide time for damage repair. There are clearly very complex interaction networks in different tissues postirradiation, and the mechanisms regulating LDR-induced methylation are not fully understood. The expression of ten-eleven translocation (TET) family proteins [28], DNA damage products [29] (such as DNA adducts 8-oxoguanine) and reactive oxygen species [30] are also believed to play crucial roles, all of which need further study.

## Supporting Information

Figure S1 Rad23b and Ddit3 methylation profile determined by BSP. The filled circle for methylated site, blank circle for unmethylated site. (A) early effects; (B) delay effects.
(TIF)

Methods S1 MeDIP-on-chip and Microarray data analysis.
(DOC)

Table S1 List of primers.
(DOC)

Table S2 GO analyses: Biological Process.
(XLS)

Table S3 GO analyses:Cellular Component.
(XLS)

Table S4 GO analyses:Molecular Function.
(XLS)

Table S5 Detected notable peaks by MeDIP-chip of the 8 candidate genes.
(DOC)
