# Supplementary figures and images for: Genome-Wide Screen of DNA Methylation Changes Induced by Low Dose X-Ray Radiation in Mice
Source: PLoS One. 2014 Mar 10;9(3):e90804. doi: 10.1371/journal.pone.0090804 (PMC3948688; doi:10.1371/journal.pone.0090804)

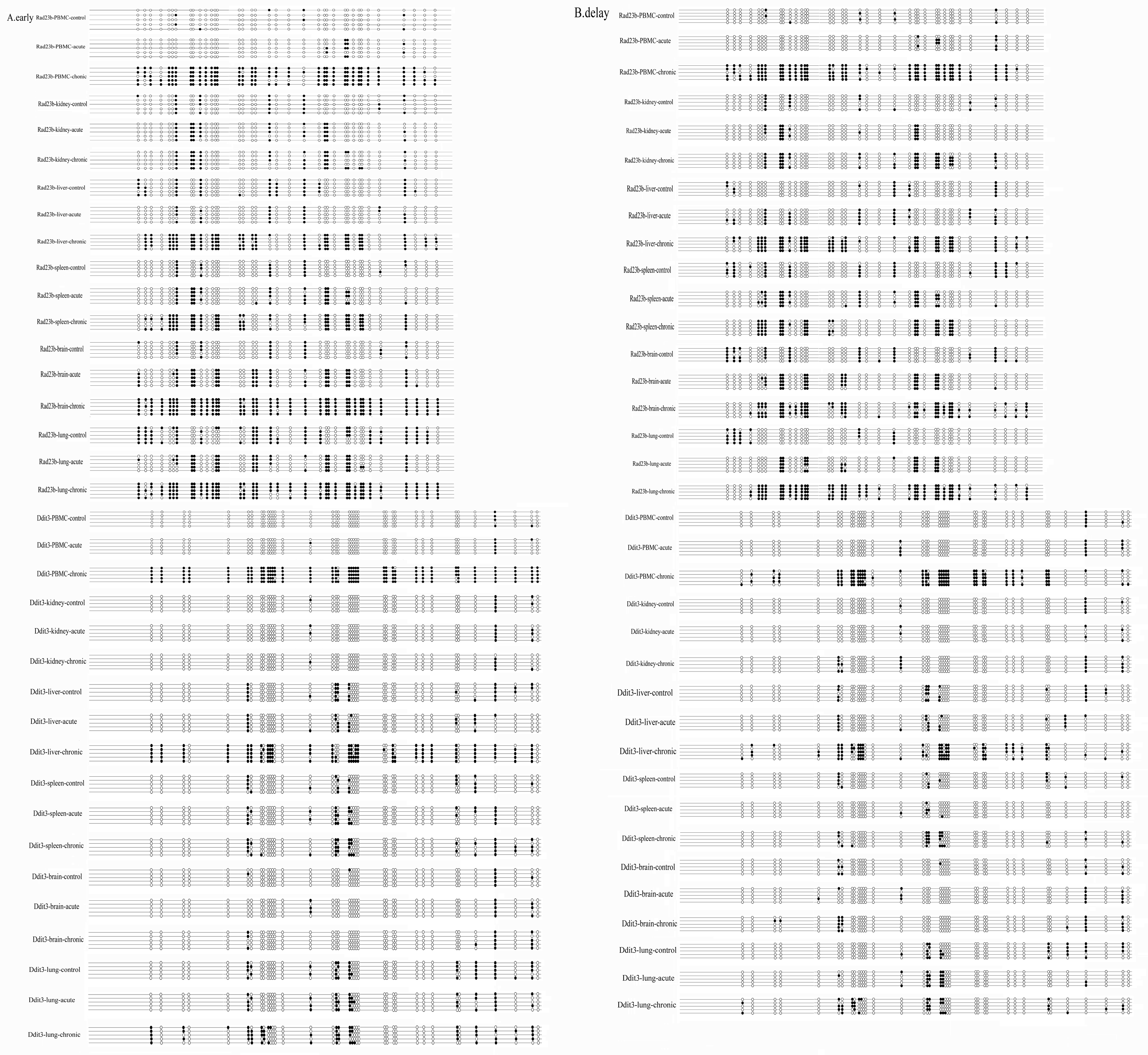

Supplement: Figure S1 — Rad23b and Ddit3 methylation profile determined by BSP. The filled circle for methylated site, blank circle for unmethylated site. (A) early effects; (B) delay effects. (TIF) [file pone.0090804.s001.tif]
