# Supplementary material for: Genome-Wide Screen of DNA Methylation Changes Induced by Low Dose X-Ray Radiation in Mice
Source: PLoS One. 2014 Mar 10;9(3):e90804. doi: 10.1371/journal.pone.0090804 (PMC3948688; doi:10.1371/journal.pone.0090804)
Supplement: Methods S1 — MeDIP-on-chip and Microarray data analysis. (DOC) [file pone.0090804.s002.doc]

*MeDIP-on-chip*

The Genomic DNA was extracted from whole blood samples collected 2h postirradiation, using QIAamp DNA Blood Mini Kit (Qiagen, Hilden, Germany) and sonicated to ~200 – 1000 bp with a Bioruptor sonicator (Diagenode). Equal DNA from each mouse within the group (5 per group) was mixtured. About 1 μg of mixtured DNA was sonicated, then used for immunoprecipitation using a mouse monoclonal anti-5-methylcytosine antibody (Diagenode). For this, DNA was heat-denatured at 94 °C for 10 min, rapidly cooled on ice, and immunoprecipitated with 1 μL primary antibody overnight at 4 °C with rocking agitation in 400 μL immunoprecipitation buffer (0.5% BSA in PBS). To recover the immunoprecipitated DNA fragments, 200 μL of anti-mouse IgG magnetic beads were added and incubated for an additional 2 hours at 4 °C with agitation. After immunoprecipitation, a total of five immunoprecipitation washes were performed with ice-cold immunoprecipitation buffer. Washed beads were resuspended in TE buffer with 0.25% SDS and 0.25mg/mL proteinase K for 2 hours at 65°C and then allowed to cool down to room temperature. MeDIP DNA was purified using Qiagen MinElute columns (Qiagen). The MeDIP-enriched DNA was amplified using a WGA kit from Sigma-Aldrich (GenomePlex® Complete Whole Genome Amplification (WGA2) kit). The amplified DNA samples were then purified with QIAquick PCR purification kit (Qiagen). For DNA labelling, the NimbleGen Dual-Color DNA Labeling Kit was used according to the manufacturer’s guideline detailed in the NimbleGen MeDIP-chip protocol (Nimblegen Systems, Inc., Madison, WI, USA). 1 μg DNA of each sample was incubated for 10 min at 98°C with 1 OD of Cy5-9mer primer (IP sample) or Cy3-9mer primer (Input sample). Then, 100 pmol of deoxynucleoside triphosphates and 100U of the Klenow fragment (New England Biolabs, USA) were added and the mix incubated at 37°C for 2 hours. The reaction was stopped by adding 0.1 volume of 0.5 M EDTA, and the labeled DNA was purified by isopropanol / ethanol precipitation. Microarrays were hybridized at 42°C during 16 to 20 h with Cy3/5 labelled DNA in Nimblegen hybridization buffer/ hybridization component A in a hybridization chamber (Nimblegen Systems, Inc.). Following hybridization, washing was performed using the Nimblegen Wash Buffer kit. For array hybridization, Roche NimbleGen's Mouse Promoter plus CpG Island array was used, which is a 3×720k format array design containing 15,980 annotated CpG islands as well as 20,404 promoters (from about -2,960bp to +740bp of the TSSs) totally covered by ~720,000 probes. Data are representative of 3 independent experiments.

*Microarray data analysis*

From the normalized log2-ratio data, a sliding-window (750bp) peak-finding algorithm provided by NimbleScan v2.5 (Roche-NimbleGen) was applied to analysis the MeDIP-chip data. A one-sided Kolmogorov-Smirnov (KS) test was used to determine whether the probes were drawn from a significantly more positive distribution of intensity log2-ratios than those in the rest of the array. Each probe received a -log10 p-value score from the windowed KS test around that probe. If several adjacent probes rised significantly above a set threshold, the region was assigned to an enrichment peak (EP). The peak data (*_peaks.gff) was generated from the p-value data (*_pvalues.gff). NimbleScan detects peaks by searching for at least 2 probes above a *P*-value minimum cutoff (-log10) of 2. Peaks within 500 bp of each other are merged. The enrichment of MeDIP signals was visualized with SignalMap version 1.9. In order to analyze the biological function that is regulated by methylation, we performed gene ontology (GO) analysis by three domains: Biological Process, Cellular Component and Molecular Function. These genes were also analyzed from the perspective of Kyoto Encyclopedia of Genes and Genomes (KEGG) pathway. The *P*-value denotes the significance of GO terms enrichment in the DE genes. The lower the *P*-value, the more significant the GO Term (*P* -value<=0.05 is recommended). The higher the Enrichment Score (ES, it equals -log10 (Pvalue)), the more significant the enrichment is.
