# Supplementary material for: Genome-Wide Screen of DNA Methylation Changes Induced by Low Dose X-Ray Radiation in Mice
Source: PLoS One. 2014 Mar 10;9(3):e90804. doi: 10.1371/journal.pone.0090804 (PMC3948688; doi:10.1371/journal.pone.0090804)
Supplement: Table S1 — List of primers. (DOC) [file pone.0090804.s003.doc]

**Supplementary Table S1**. List of primers

| **Gene Name** | **Primer Sequence** | **Tm (℃)** | **Product (bp)** |
| --- | --- | --- | --- |
| GAPDH | F: 5′- AACTTTGGCATTGTGGAAGG -3′  R: 5′- ACA CATTGGGGGTAGGAACA -3′ | 56 | 223 |
| DNMT1(qPCR) | F:5′-CCCAAAGAAGGATCCTGTGA-3’  R:5′-CTTGATGTCTGCCTCGTTGA-3′ | 55 | 169 |
| MBD2(qPCR) | F:5′- CTGGCA AGATACCTGGGAAA-3′ | 53 | 104 |
|  | R:5′- TTCCGGAGTCTCTGCTTGTT -3′ |  |  |
| Rad23b(qPCR) | F: 5’CAGCTGACAGTACACCAGGA3’ | 58 | 151 |
|  | R: 5’GCTCTCAGGGCTGCAATTAC3’ |  |  |
| Ddit3(qPCR) | F: 5’ACGGAAACAGAGTGGTCAGT3’ | 58 | 226 |
|  | R: 5’AGACAGACAGGAGGTGATGC3’ |  |  |
| Rad23b(MeDIP-PCR) | F:5’GCAACCGCCTAATATCTACCAC3’ | 60 | 220 |
|  | R:5’ACATCGCCAGTCGAGCATG3’ |  |  |
| Tdg(MeDIP-PCR) | F:5’TTTGCCACCACGCATAATACATC3’  R:5’GACCGCCGCTTAGCCTTTC3’ | 60 | 145 |
| Ccnd1(MeDIP-PCR) | F:5’CTGCGAAGTGGAGACCATCC3’  R:5’TTGAGGAGATTGGTGTCAGGGT3’ | 60 | 51 |
| Ddit3(MeDIP-PCR) | F:5’CGTGACCCAAAGCCACTTC3’  R:5’ACCAATCAGAAAGCGGCAC3’ | 60 | 143 |
| Llgl1(MeDIP-PCR) | F:5’GGCAAAGTAGTGATCCGTGAA3’  R:5’CTTGTAGTCCGAAGGTGGGTC3’ | 60 | 197 |
| Rasl11a(MeDIP-PCR) | F:5’CCTCCAGGACACGGCAAATAC3’  R:5’TGCCCAGGCTCTTGGATTTTA3’ | 60 | 235 |
| Tbx2(MeDIP-PCR) | F:5’CCGCAGCCTCCCTCTGAAGT3’  R:5’CCGATCTGACCCGCCGTAA3’ | 60 | 243 |
| Slc6a15(MeDIP-PCR) | F:5’CCCCAGATCCACCCAATATC3’  R:5’ACCCATGACGCAGAGTCCA3’ | 60 | 90 |
| Rad23b (BSP) | F:5’AGGTTTYGGTTTAGGTATTATG 3’ | 57 | 353 |
|  | R:5’CCTAAATCTCCCTCCCTC 3’ |  |  |
| Ddit3 (BSP) | F:5’AGGGAAAATGGGAGTTTTTTAT 3’ | 60 | 448 |
|  | R:5’CCAAAAACCTACCAATCAAAA 3’ |  |  |
