# Supplementary material for: Genome-Wide Screen of DNA Methylation Changes Induced by Low Dose X-Ray Radiation in Mice
Source: PLoS One. 2014 Mar 10;9(3):e90804. doi: 10.1371/journal.pone.0090804 (PMC3948688; doi:10.1371/journal.pone.0090804)
Supplement: Table S5 — Detected notable peaks by MeDIP-chip of the 8 candidate genes. (DOC) [file pone.0090804.s007.doc]

**Supplementary Table S5. Detected notable peaks by MeDIP-chip of the 8 candidate genes**

| **Peak ID** | **Peak Score** | **PeakM value** | **Gene Name** | **Promoter_**  **Classfication** | **Chromosome** | **Function** |
| --- | --- | --- | --- | --- | --- | --- |
| 27421 | 2.17 | 1.83 | Rad23b | HCP | Chr4 | nucleotide-excision repair, DNA damage recognition |
| 21631 | 2.06 | 1.12 | Tdg | HCP | Chr10 | DNA repair |
| 30293 | 2.79 | 0.79 | Ccnd1 | ICP | Chr7 | G1/S transition of mitotic cell cycle |
| 21758 | 2.61 | 0.76 | Ddit3 | HCP | Chr10 | cell cycle arrest, apoptotic process, Wnt receptor signaling pathway |
| 22054 | 2.71 | 1.30 | Llgl1 | HCP | Chr11 | Hippo signaling pathway, organism-specific biosystem |
| 28784 | 2.22 | 1.55 | Rasl11a | HCP | Chr5 | GTP catabolic process |
| 22316 | 2.42 | 1.78 | Tbx2 | ICP | Chr11 | Heart development, organism-specific biosystem |
| 21684 | 2.15 | 1.49 | Slc6a15 | ICP | Chr10 | Amino acid, ion, leucine, meutral amino acid, and proline transport. |
